# Supplementary material for: Minimum days estimation for reliable dietary intake information: findings from a digital cohort
Source: Eur J Clin Nutr. 2025 Jul 24;79(10):1007–17. doi: 10.1038/s41430-025-01644-8 (PMC12537485; doi:10.1038/s41430-025-01644-8)
Supplement: Supplementary file 1 — Supplementary File [file 41430_2025_1644_MOESM1_ESM.docx]

**Minimum Days Estimation for Reliable Dietary Intake Information: Findings from a Digital Cohort**

*Rohan Singh^1^, Mathieu Théo Eric Verest^1^, Marcel Salathé^1^*

*^1^ Digital Epidemiology Lab, School of Life Sciences, School of Computer and Communication Sciences, EPFL, Switzerland*

Correspondence to: marcel.salathe@epfl.ch

**Supplementary Figures**

**
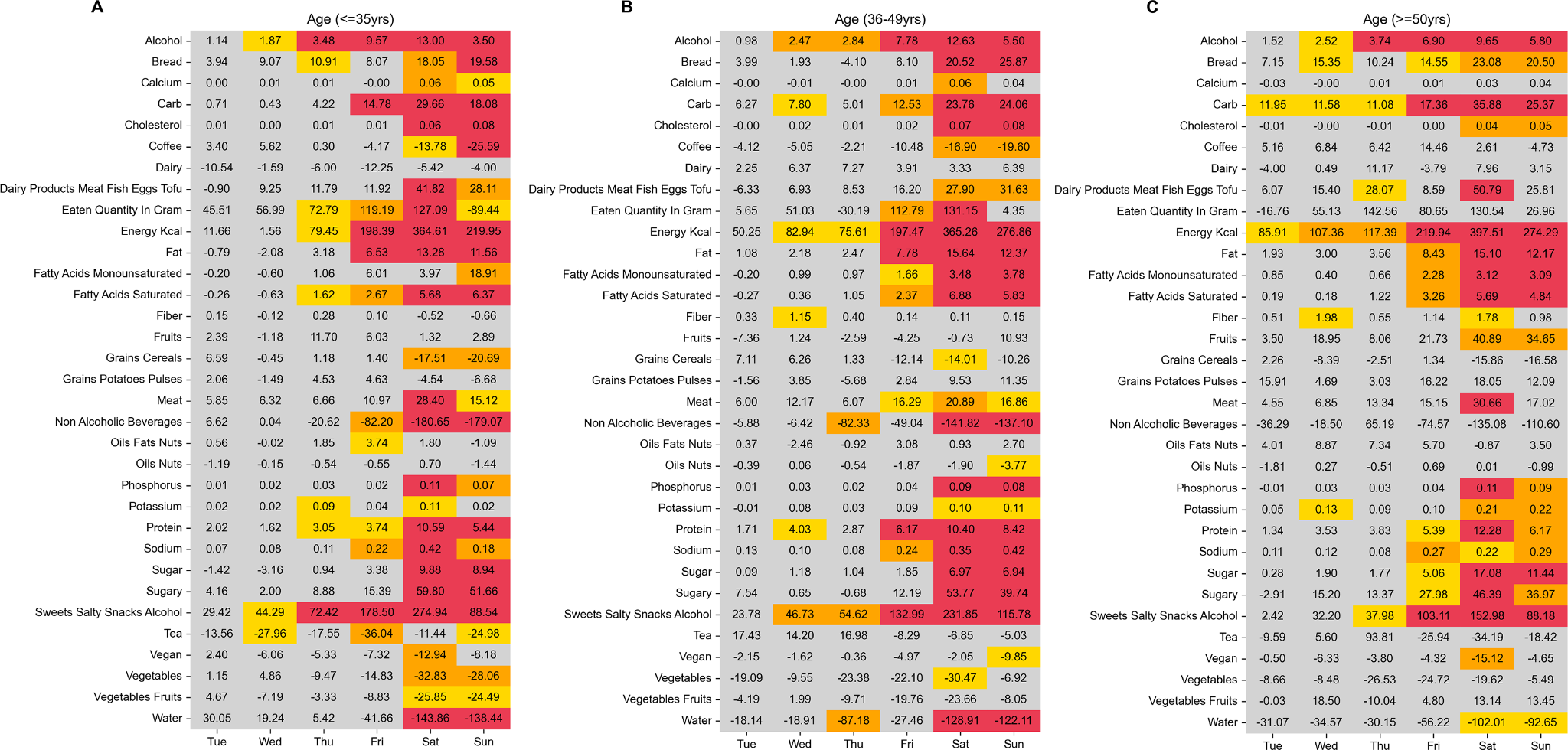
**

**Supplementary Figure 1:** Coefficients and p-values of Linear Mixed Models for various nutrients across different days of the week of the “Food & You” participants, subsetted by age groups. The x-axis represents the days of the week (Tuesday to Sunday), with Monday as the reference day. The cells display the coefficients, indicating the change in nutrient intake compared to Monday. The color of the cells represents the statistical significance of the coefficients: red for p < 0.001, orange for p < 0.01, yellow for p < 0.05, and gray for p ≥ 0.05.


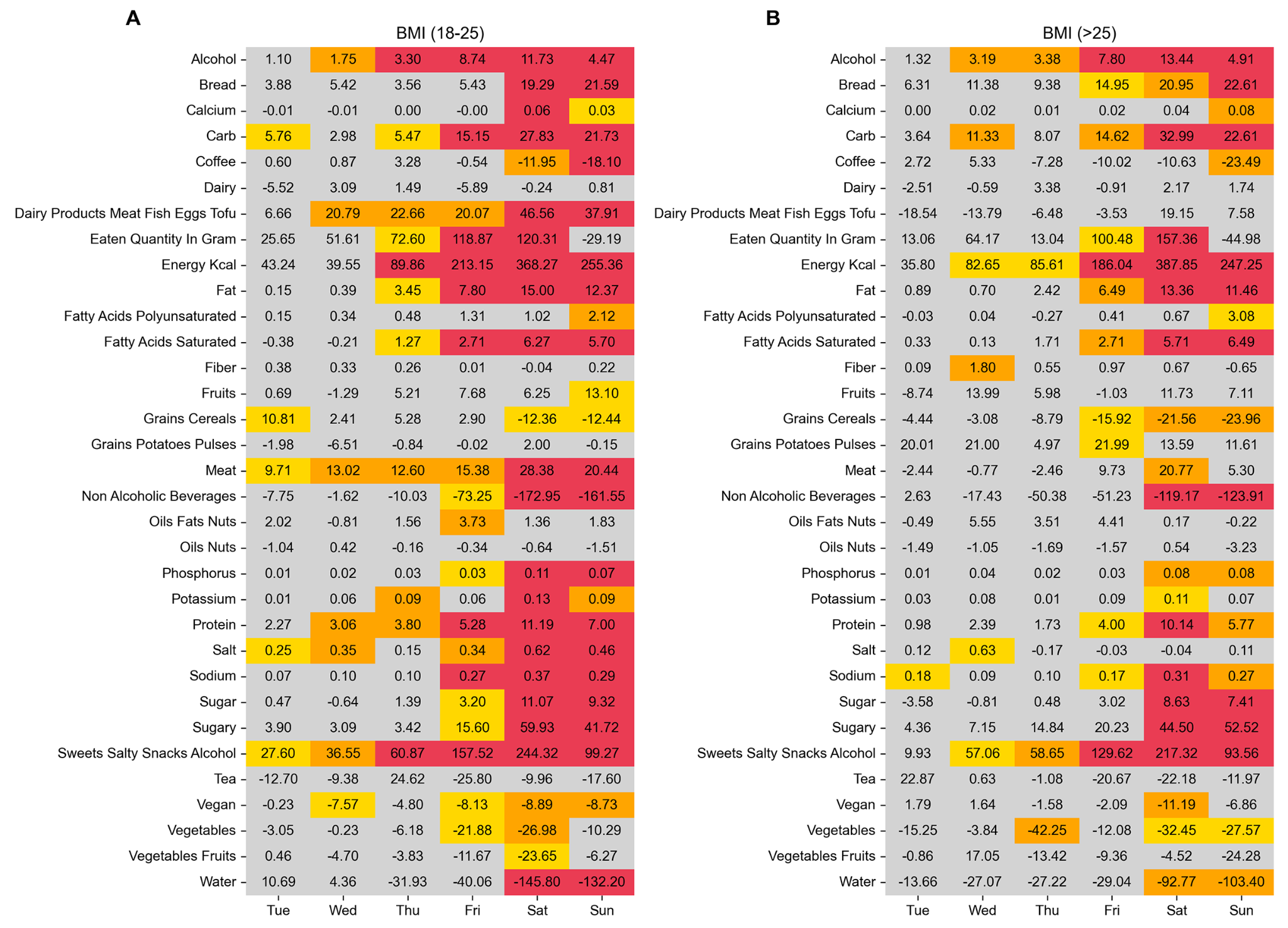
**Supplementary Figure 2:** Coefficients and p-values of Linear Mixed Models for various nutrients across different days of the week of the “Food & You” participants, subsetted by BMI. The x-axis represents the days of the week (Tuesday to Sunday), with Monday as the reference day. The cells display the coefficients, indicating the change in nutrient intake compared to Monday. The color of the cells represents the statistical significance of the coefficients: red for p < 0.001, orange for p < 0.01, yellow for p < 0.05, and gray for p ≥ 0.05.


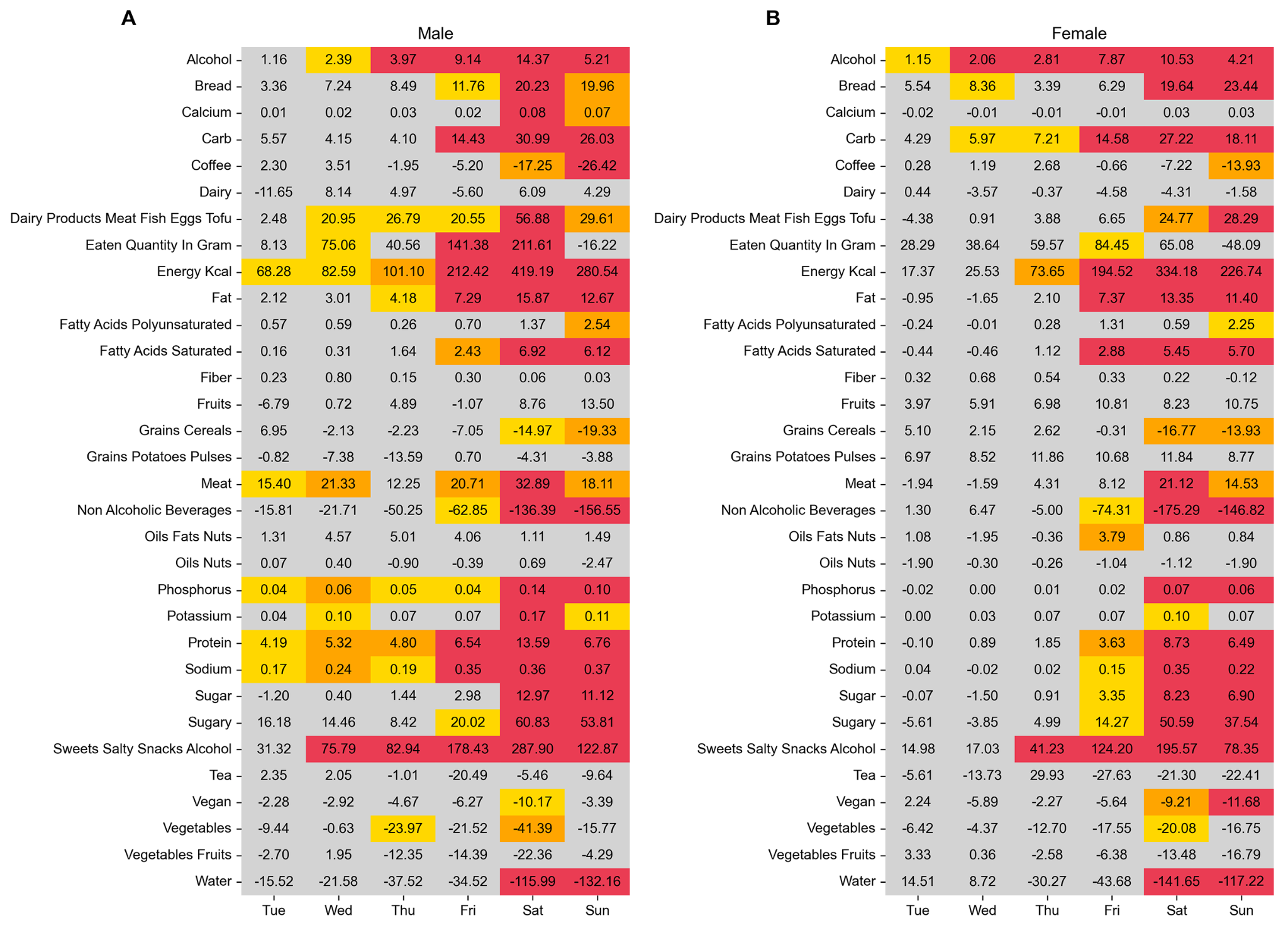


**Supplementary Figure 3:** Coefficients and p-values of Linear Mixed Models for various nutrients across different days of the week of the “Food & You” participants, subsetted by sex. The x-axis represents the days of the week (Tuesday to Sunday), with Monday as the reference day. The cells display the coefficients, indicating the change in nutrient intake compared to Monday. The color of the cells represents the statistical significance of the coefficients: red for p < 0.001, orange for p < 0.01, yellow for p < 0.05, and gray for p ≥ 0.05.


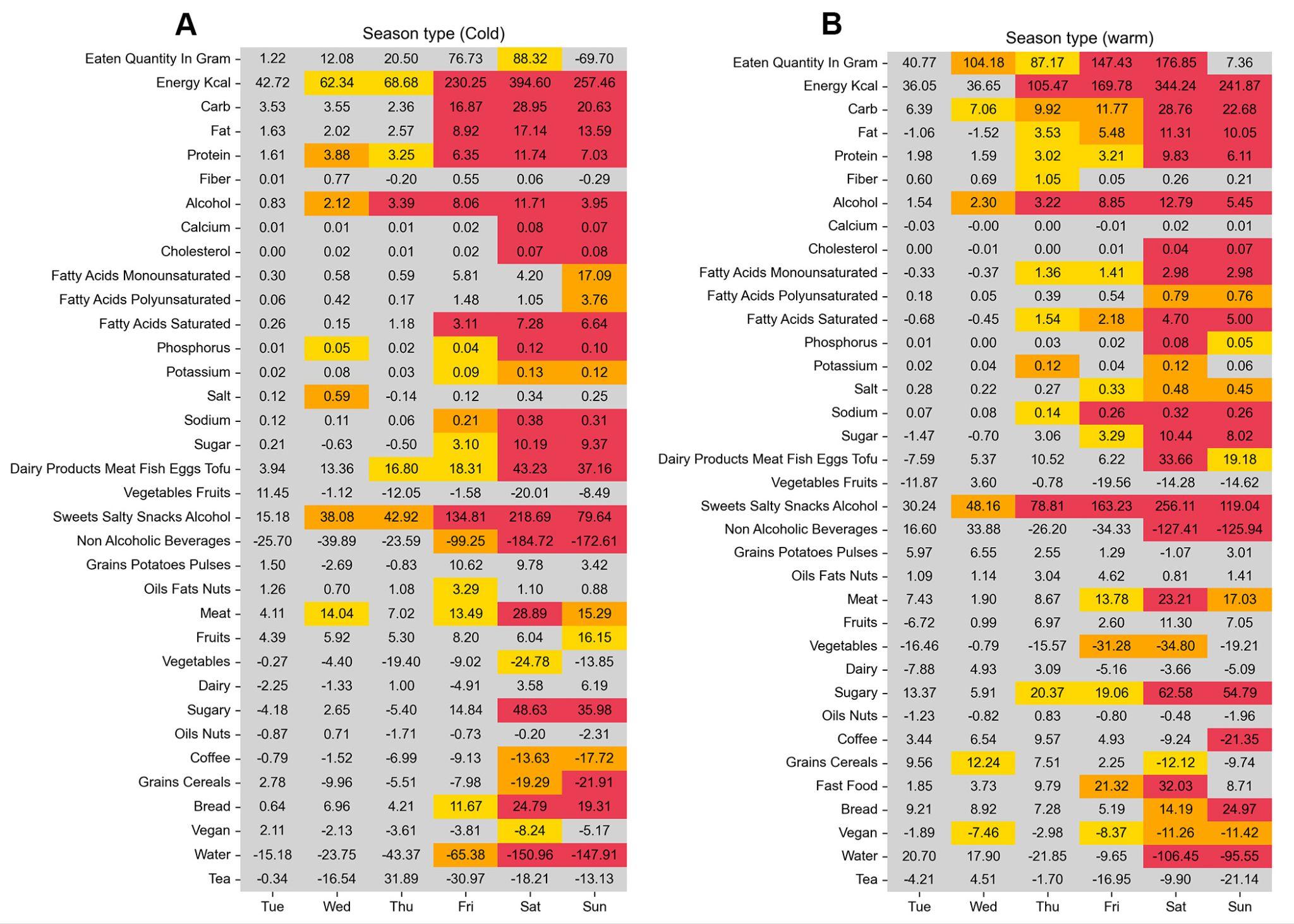


**Supplementary Figure 4:** Coefficients and p-values of Linear Mixed Models for various nutrients across different days of the week of the “Food & You” participants, subsetted by season, i.e., months are categorized into cold (November through April) and warm (May through October) seasons. The x-axis represents the days of the week (Tuesday to Sunday), with Monday as the reference day. The cells display the coefficients, indicating the change in nutrient intake compared to Monday. The color of the cells represents the statistical significance of the coefficients: red for p < 0.001, orange for p < 0.01, yellow for p < 0.05, and gray for p ≥ 0.05.


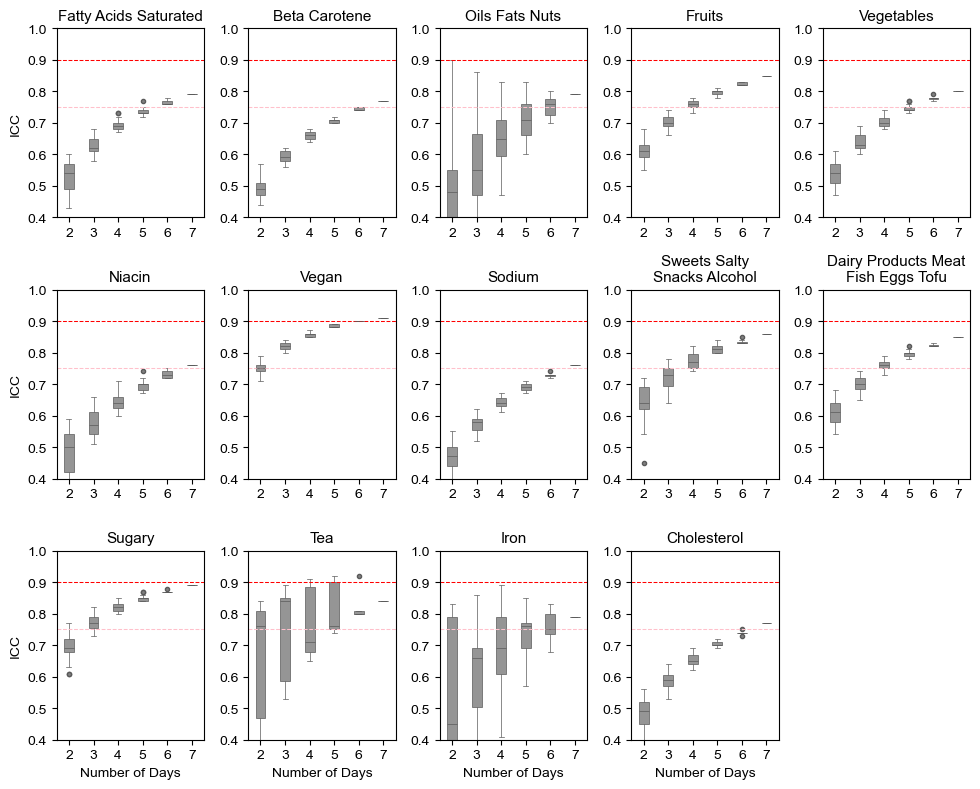


**Supplementary Figure 5:** Distribution of ICC values across different numbers of days for various nutrient intakes for which the ICC value for at least a single combination exceeded 0.75. The x-axis represents the number of days, while the y-axis represents the ICC scores. Each subplot corresponds to a different nutrient, with boxplots indicating the ICC scores for combinations of days for each minimum number of days on the x-axis. The horizontal red lines in each subplot denote the ICC threshold of 0.75 (good reliability) and 0.9 (excellent reliability). Boxplot colors represent different nutrient groups: blue for micronutrients, red for food groups, and green for macronutrients. Note that ICC does not generally reach 1 due to inherent within-subject variability.


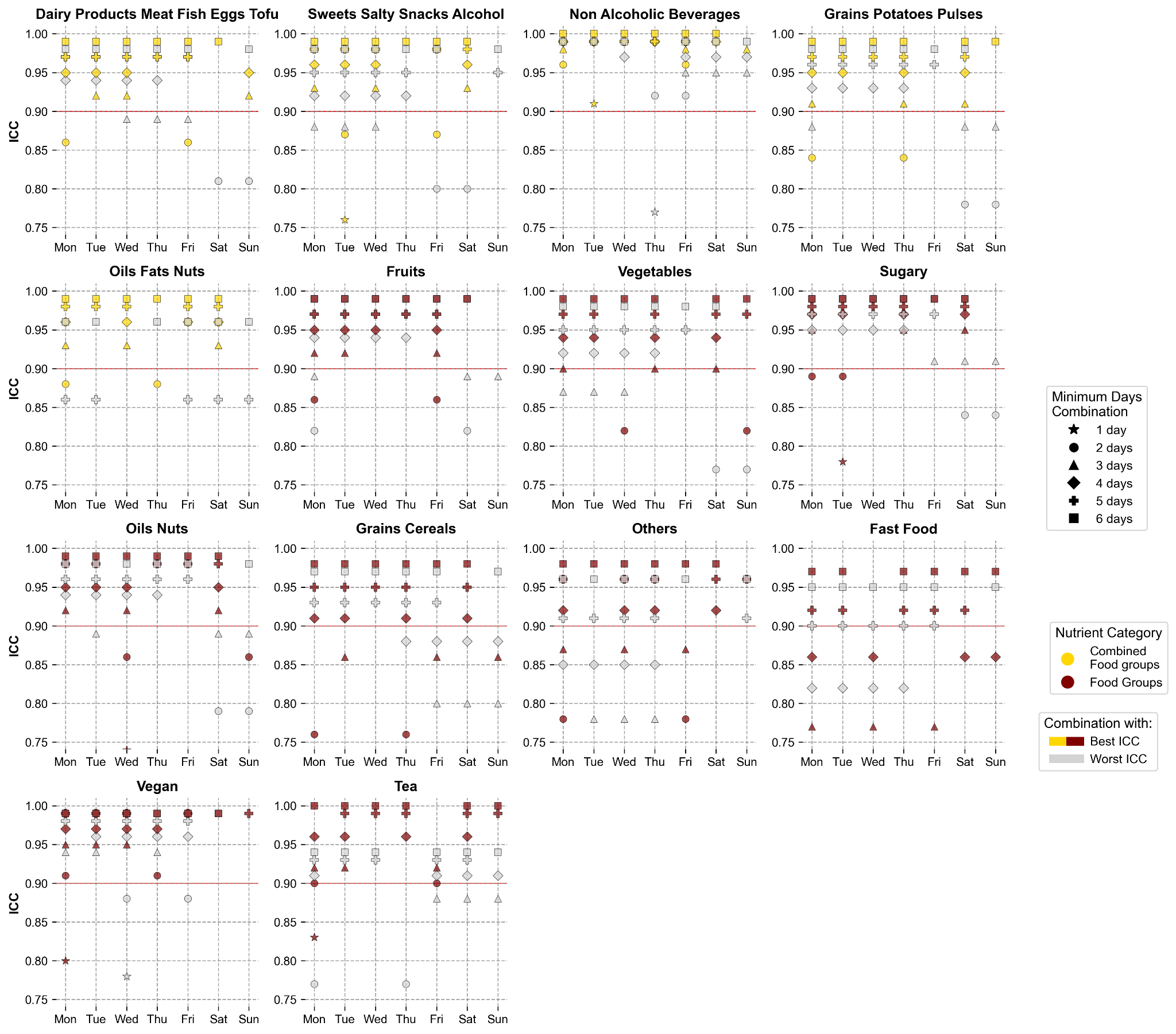


**Supplementary Figure 6:** Best and worst day combinations at different minimum days for reliable dietary assessment of different food groups. For each food group and at each number of days, the day combinations which yielded the highest (in color) and lowest ICC scores (in gray) are shown. The plot is ranged between ICC values of 0.75 to 1.0, with the ICC reliability threshold at 0.9 shown as a red line - points lying below this range are not shown.


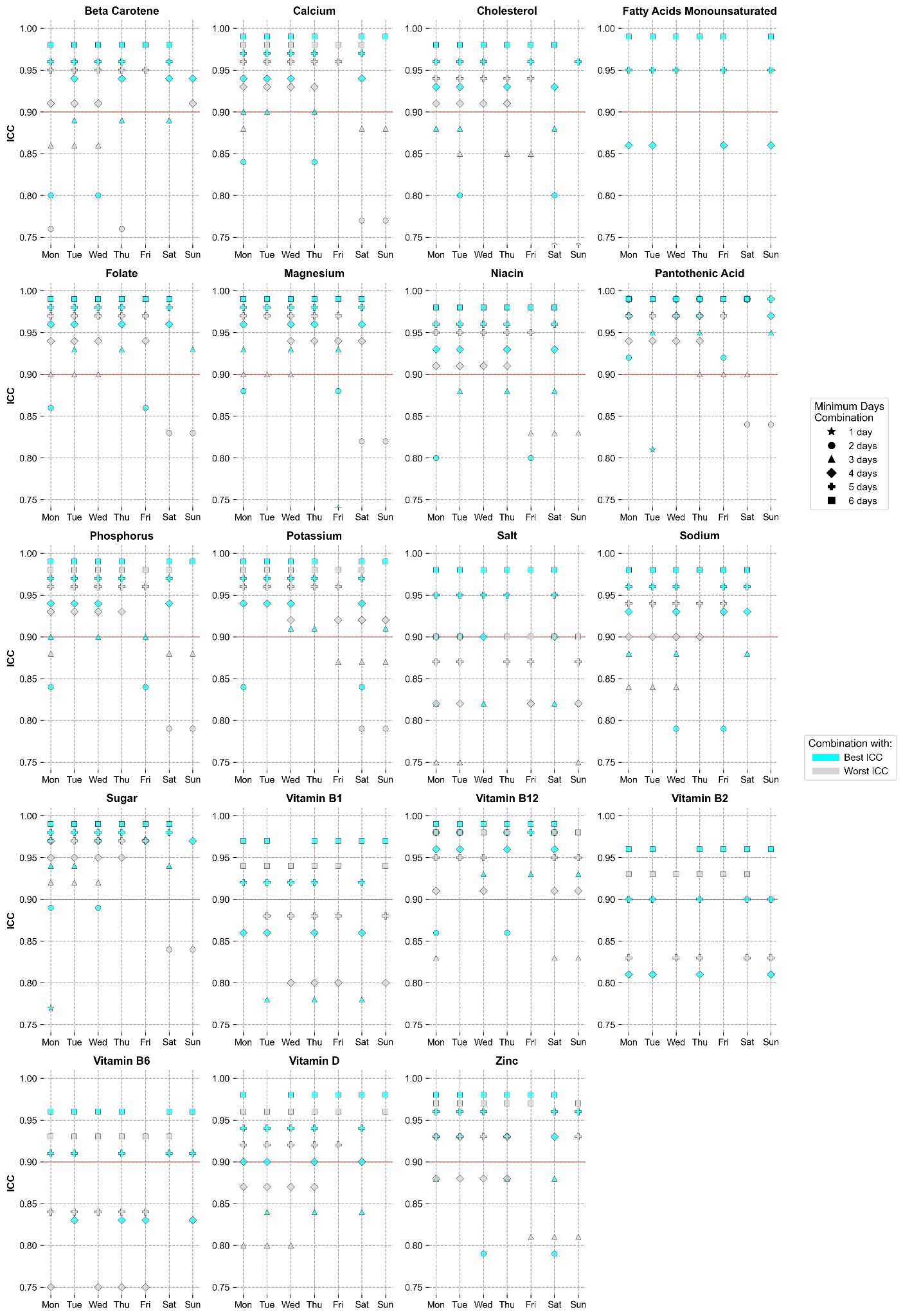


**Supplementary Figure 7:** Best and worst day combinations at different minimum days for reliable dietary assessment of different micronutrients. For each nutrient and at each number of days, the day combinations which yielded the highest (in color) and lowest ICC scores (in gray) are shown. The plot is ranged between ICC values of 0.75 to 1.0, with the ICC reliability threshold at 0.9 shown as a red line - points lying below this range are not shown.
